# Diagnostic AI Modeling and Pseudo Time Series Profiling of AD and PD Based on Individualized Serum Proteome Data

**DOI:** 10.3389/fbinf.2021.764497

**Published:** 2021-10-22

**Authors:** Jianhu Zhang, Xiuli Zhang, Yuan Sh, Benliang Liu, Zhiyuan Hu

**Affiliations:** ^1^ Fujian Provincial Key Laboratory of Brain Aging and Neurodegenerative Diseases, School of Basic Medical Sciences, Fujian Medical University, Fuzhou, China; ^2^ CAS Key Laboratory of Standardization and Measurement for Nanotechnology, CAS Key Laboratory for Biomedical Effects of Nanomaterials and Nanosafety, CAS Center for Excellence in Nanoscience, National Center for Nanoscience and Technology of China, Beijing, China; ^3^ China National Center for Bioinformation, Beijing, China; ^4^ Key Laboratory of Genomic and Precision Medicine, Beijing Institute of Genomics, Chinese Academy of Sciences, Beijing, China; ^5^ School of Nanoscience and Technology, Sino-Danish College, University of Chinese Academy of Sciences, Beijing, China; ^6^ School of Chemical Engineering and Pharmacy, Wuhan Institute of Technology, Wuhan, China

**Keywords:** alzheimer’s disease, parkinson’s disease, mild cognitive impairment, artificial intelligence, predictive diagnostics

## Abstract

**Background:** Parkinson’s disease (PD), Alzheimer’s disease (AD) are common neurodegenerative disease, while mild cognitive impairment (MCI) may be happened in the early stage of AD or PD. Blood biomarkers are considered to be less invasive, less cost and more convenient, and there is tremendous potential for the diagnosis and prediction of neurodegenerative diseases. As a recently mentioned field, artificial intelligence (AI) is often applied in biology and shows excellent results. In this article, we use AI to model PD, AD, MCI data and analyze the possible connections between them.

**Method:** Human blood protein microarray profiles including 156 CT, 50 MCI, 132 PD, 50 AD samples are collected from Gene Expression Omnibus (GEO). First, we used bioinformatics methods and feature engineering in machine learning to screen important features, constructed artificial neural network (ANN) classifier models based on these features to distinguish samples, and evaluated the model’s performance with classification accuracy and Area Under Curve (AUC). Second, we used Ingenuity Pathway Analysis (IPA) methods to analyse the pathways and functions in early stage and late stage samples of different diseases, and potential targets for drug intervention by predicting upstream regulators.

**Result:** We used different classifier to construct the model and finally found that ANN model would outperform the traditional machine learning model. In summary, three different classifiers were constructed to be used in different application scenarios, First, we incorporated 6 indicators, including EPHA2, MRPL19, SGK2, to build a diagnostic model for AD with a test set accuracy of up to 98.07%. Secondly, incorporated 15 indicators such as ERO1LB, FAM73B, IL1RN to build a diagnostic model for PD, with a test set accuracy of 97.05%. Then, 15 indicators such as XG, FGFR3 and CDC37 were incorporated to establish a four-category diagnostic model for both AD and PD, with a test set accuracy of 98.71%. All classifier models have an auc value greater than 0.95. Then, we verified that the constructed feature engineering filtered out fewer important features but contained more information, which helped to build a better model. In addition, by classifying the disease types more carefully into early and late stages of AD, MCI, and PD, respectively, we found that early PD may occur earlier than early MCI. Finally, there are 24 proteins that are both differentially expressed proteins and upstream regulators in the disease group versus the normal group, and these proteins may serve as potential therapeutic targets and targets for subsequent studies.

**Conclusion:** The feature engineering we build allows better extraction of information while reducing the number of features, which may help in subsequent applications. Building a classifier based on blood protein profiles using deep learning methods can achieve better classification performance, and it can help us to diagnose the disease early. Overall, it is important for us to study neurodegenerative diseases from both diagnostic and interventional aspects.

## Introduction

Neurodegenerative diseases are nervous system disorders that manifest as a progressive loss of function or structure of neurons, including the death of neurons. The most extensively studied neurodegenerative diseases are Alzheimer’s disease (AD) and Parkinson’s disease (PD). AD, the leading cause of dementia (Long and Holtzman, 2019), is a disease associated with cognitive impairment, presents with learning, language, and memory impairment (McKhann et al., 2011). PD is a complex disorder of the brain system that affects not only movement, such as rigidity, bradykinesia and tremor, but also cognition. Mild cognitive impairment (MCI) is characterized by persistent memory problems and is considered an asymptomatic pre-dementia of AD, a non-motor symptom that occurs early in PD (Albert et al., 2011; Poewe et al., 2017). It is important to distinguish MCI status because some studies suggest that MCI may lead to the development of AD, and PD with MCI may have a higher risk of developing Parkinson’s disease dementia (PDD) (Pagani et al., 2017; Saredakis et al., 2019).

When an individual is diagnosed with AD or PD, pathological damage in the brain has actually occurred for some time, which is irreversible (Gaig and Tolosa, 2009; Petersen, 2009). Therefore, early diagnosis of both disease is very necessary, blood biomarkers have got more attention due to more convenient, less costly, and less risky sampling. Eric P. Nagele found that differentially expressed proteins (DEPs) in human blood can better distinguish AD, PD, and MCI from normal samples respectively (Nagele et al., 2011; Han et al., 2012; DeMarshall et al., 2016). For example, the accuracy of distinguishing AD from normal samples was 93.4%, while the accuracy of distinguishing PD from normal samples was 97.1%. Although there have been many studies in this field, few researchers have studied MCI, PD and AD together despite their potential association, and we believe that a combined study would help to more fully understand the relationship. In the process of data modeling, traditional machine learning researchers usually use feature engineering to process the data and rarely use bioinformatics methods, while the opposite is true for traditional bioinformatics researchers, which may not yield optimal results. We innovative combine bioinformatics screening differential protein methods with machine learning feature engineering for data processing and model building, and achieve better results. In this paper, based on serum protein expression profiles, we build a model to distinguish AD, MCI, PD, and CT samples and search possible biological phenomenon and drug targets.

## Materials and Methods

### Data Sources and Preprocessing

We downloaded multiple protein expression profiles from the GEO database (https://www.ncbi.nlm.nih.gov/geo/), including GSE29654, GSE62283, GSE74763. These datasets were generated by the Invitrogen ProtoArray V5.0 platform. Since there were some duplicate samples in these three datasets, we kept the duplicate samples with the earlier upload time. We also removed samples from the same institution but with a sample size of less than 3. Finally, the number of samples in each category, as shown in Table 1.

**TABLE 1:** Overview of the collected data.

**Group**	**Sample numbers**	**Age [Median (Range)]**	**Sex (% male)**
Control	156	56.50 (19–86)	56.41
Mild cognitive impairment	50	73.00 (55–91)	58.00
Alzheimei’s disease	50	79.00 (61–97)	43.47
Parkinson’s disease	132	66.00 (37–88)	57.69

The format of raw data is GPR, we use the R package PPA to load it, then normalized the data with the robust linear model (RLM) method, which is the standard intra-slice method capable of ignoring the effect of isolated points, allowing a good fit of the regression line. Finally, common probes were extracted and expression profiles were merged. We standardized the data by the following method, for each gene in each sample, calculated the ratio of that gene to the total gene expression in the sample and multiplied the ratio by 1 million as the final expression value. The formula is as follows, where the data is a two-dimensional table with i rows and j columns, the row stores the protein, j represents the sample, x^ij^ represents the expression value of the ith protein of the jth sample, and x^ij’^ represents the standardized data.

$$x_{ij}^{'}=\left(\frac{x_{ij}}{\sum_{i=1}^{n}x_{ij}}\right)*1000000$$

### Model of Machine Learning and Deep Learning

Four machine learning classifiers were used in the construction of the model, including naive Bayes (NB) (Zhang, 2004), k-nearest neighbor (KNN) (Troyanskaya et al., 2001), decision tree (DT) (Breiman et al., 2017), random forest (RF) (Breiman, 2001), and a deep learning classifier, ANN. Machine learning models are stored in scikit-learn 0.23.1 and Python 3.8.3 (Pedregosa et al., 2011). When building the ANN, we use the Keras 2.4.3 module. The default parameters of sklearn and keras basic classifier are modified during model training, where KNN (n_neighbors = 3), DT (criterion: “gini”, splitter: “best,” min_samples_split: 2, min_samples_leaf: 1), random forest (n_estimators: 100,criterion: “gini,”max_features: "auto",etc), ANN (activation_relu: “relu,” optimizers: “adam,” batch_size: 64, etc). All model parameters can be found in the https://github.com/zhxiuli/AI.git.

### Model Construct and Model Evaluation

The DEPs were identified using the limma Bioconductor package in R (Version 3.6.3) (Ritchie et al., 2015). First, we extracted the union of DEPs between pairs of categories as the initial features. According to the variance of these initial features, proteins with variance less than a quarter of the population were eliminated. Then correlation was used to remove proteins with correlation coefficient greater than 0.7 with other proteins. Finally, we used an SVM-based model to extract the top N features that are most important for model construction (Cortes and Vapnik, 1995; Guyon et al., 2002). In the process of selecting the most important features, we set the step size to 1, i.e., we use an iterative approach to eliminate features one by one until the performance is optimal. For all the classifiers, the ratio of the training set, validation set, the test set is 3:1:1. The training set is used to train the model, the validation set selects the optimal model parameters, and the test set to evaluate model performance. We use micro-AUC method to analyze the AUC of multi-label classification. Assume the original data is n samples with m columns of features. The basic idea is to binarize the original labels of each sample, so that the samples can also get the format of (n,m) (the position corresponds to 1 and the rest to 0), and then the probability matrix and label matrix of the multi-label are expanded by rows respectively as a way to calculate the AUC value of the binary classification.

### IPA and Protein Phase Separation

IPA is a cloud-based integrated biological pathway analysis commercial software developed for biologists, in which the software analysis data is manually extracted from major professional journals and magazines by life science experts, mainly used in life science research. The IPA was used for biological analysis, including canonical pathway analysis, disease and function, upstream regulators. A threshold of −log 10(P-value) > 1.3 was used to indicate statistical significance, and a Z-score > 0 was defined as active, otherwise as inhibited. For protein phase separation analysis, we uploaded the sequences of the proteins to the PLAAC (http://plaac.wi.mit.edu/) to get a phase separation scores (Lancaster et al., 2014).

## Results

### Development of Individualized Diagnostic Models and Analysis Process for AD and PD Patients

**FIGURE 1:**
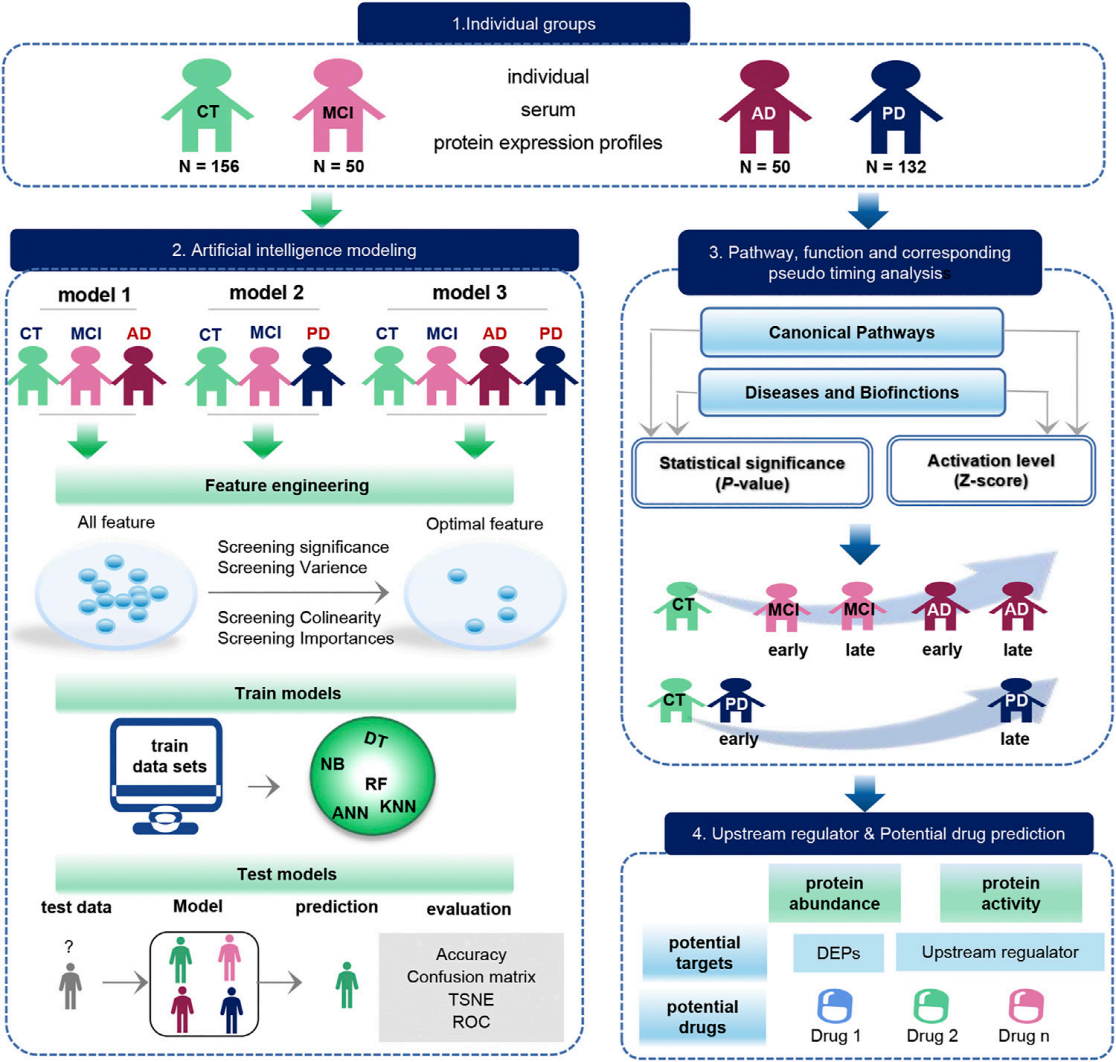
Schematic diagram of the workflow of this study. The original data set was downloaded from the GEO database, which included 156 CT samples, 50 MCI samples, 50 AD samples, and 132 PD samples. The blue arrow represents the process of exploring the biological function of the data, and the green arrow represents the modeling process performed on the data. Biological function studies how serum molecules affect the body’s cells, tissues and organs across the board as they flow with the blood, including differences in proteins between groups, as well as pathways, functions, pseudo-timing analysis, and upstream regulators and potential drugs. Data modeling constructs three models with the same process. The construction process is as follows. We first use feature engineering to extract important features, then use different machine learning model to training data, finally evaluate the classification efficiency of the model.

Based on serum protein expression profiles, we construct three individual disease diagnostic models useing artificial intelligence. In addition, biological pathways, functional, upstream factors, and pseudo-time information between diseases were mined (Figure 1). 388 serum protein expression profiles were downloaded from the GEO database, containing 156 Control samples (CT), 50 MCI, 50 AD, and 132 PD samples. On the one hand, the optimal feature for constructing the model were first filtered based on the significance, variance, colinearity, and importance. Then different classifier models are trained using the optimal features, including random forest (RF), Decision Tree (DT), and Navie Bayes (NB), Artificial neural network (ANN), k-Nearest Neighbor (KNN). The trained models were applied to the test set to observe the classification effects (accuracy, confusion matrix, ROC) and feature effects (TSNE) of the model. On the other hand, we analyzed the pathways and functions between disease and normal samples, and then analyzed the possible order of disease occurrence. Finally, we analyzed upstream regulators and possible drug targets.

### The AI Model for the Diagnosis of AD, MCI and CT Based on 6 Serum Protein Markers

**FIGURE 2:**
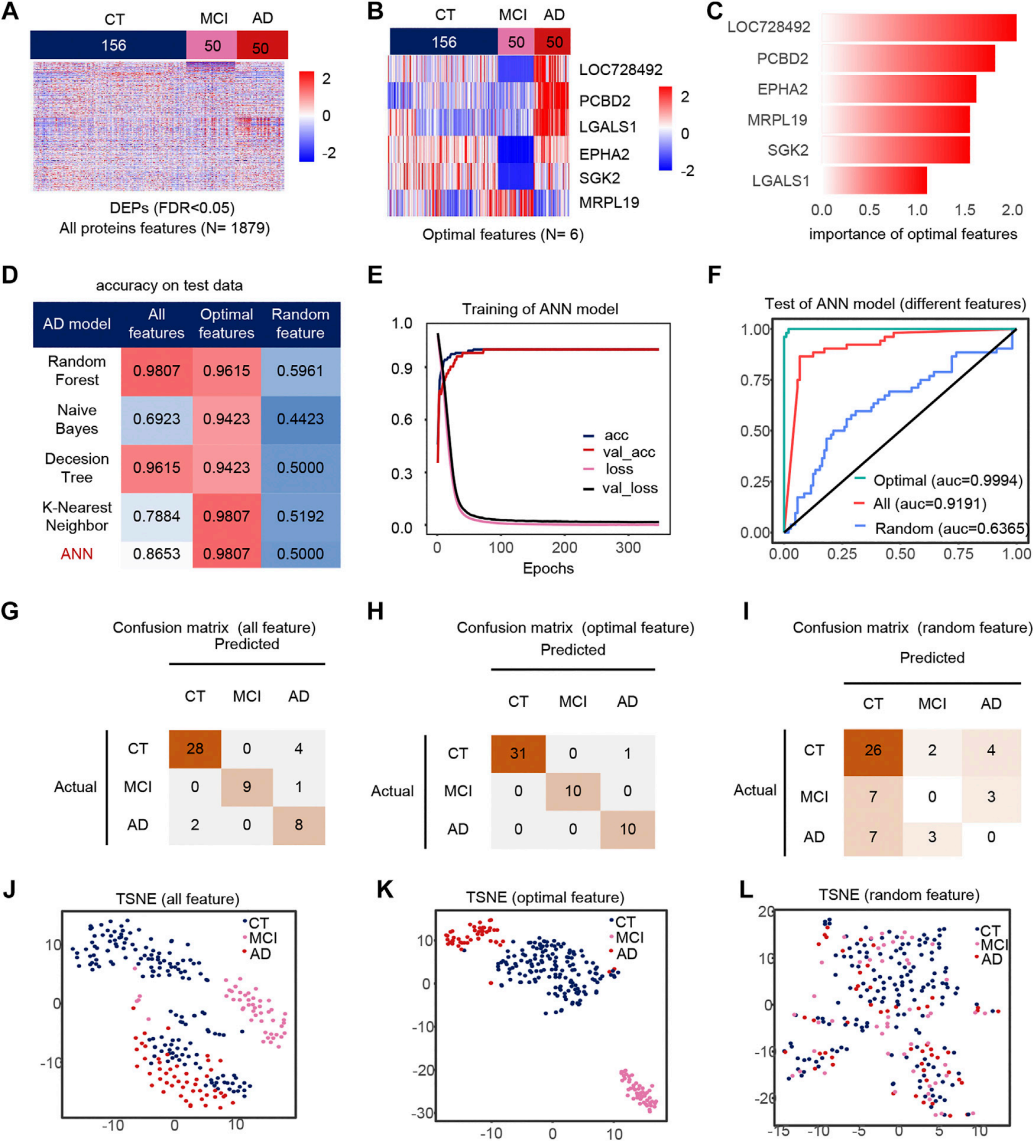
Model 1(CT, MCI, AD) feature engineering and model evaluation. **(A)** Heat map of all features in the model. **(B)** Expression profile of the optimal features in the data set. c) Importance of the optimal features filtered after feature engineering. **(D)** Accuracy of different classifiers on different features. **(E)** Accuracy and loss curves during training of the ANN model. **(F)** AUC of the ANN model when using different features. **(G–I)** Confusion matrix corresponding to all features, optimal features, and random features in the ANN model. **(J–L)** TSNE consisting of all features, optimal features, and random features.

AD, CT, and MCI samples were extracted from the dataset, and 1879 DEPs between the AD, CT, and MCI were detected (Figure 2A). When constructing the feature engineering, we observed the following principles: 1) Features with small variance have little impact on the classifier. 2) Highly correlated features may lead to covariance problems in the model. 3) A few important features are sufficient to represent the whole range of features. After variance, correlation and importance screening (Supplementary Figures S1A,B), six features were finally obtained, containing *L*O*C728492*, *PCBD2*, *EPHA*2, *MRPL19*, *SGK2*, *LGALS1*. These six optimal features were expressed significantly differently among the groups, and their importance was shown in the figure (Figures 2B,C).

We use different classifiers (KNN, RF, ANN, NB, DT) and different features (optimal features, random features, all features) to build models. In the end, the optimal features can achieve similar or even better classification performance than all features, and this is not due to randomness (Figure 2D). The accuracy and loss curves of these six features during ANN model training (Figure 2E) show that we stopped the training when the model was stable. The micro-AUC for the optimal features was 0.9994, higher than 0.9191 for all features and 0.6385 for random features (Figure 2F). The accuracy of the model was greater than 0.95 in all three test sets (Figures 2G–I), and their AUC in the test set are shown in the Supplementary Figures S4A–C. The accuracy of the model in all test set was 98.07%, where MCI and AD classification being completely correct, outperforming all features and random features (Figures 2J–L). Compared to all features and random features, the optimal features can distinguish samples well (Figures 2J–L). The above results show that the optimal features selected after feature engineering help to improve the performance and simplify the model. We defined 0 for the CT, 0.5 for the MCI and 1 for the AD sample to analyze the correlation between the optimal features and disease progression. Most features were positively correlated with the severity of cognitive loss, except for MRPL19. EPHA2 is a neuroinflammatory factor (Supplementary Figure S1C), which may indicate that the neuroinflammatory pathway in which EPHA2 resides is closely related to the progression of AD.

### The AI Model for the Diagnosis of PD, MCI and CT Based on 15 Serum Protein Markers

**FIGURE 3:**
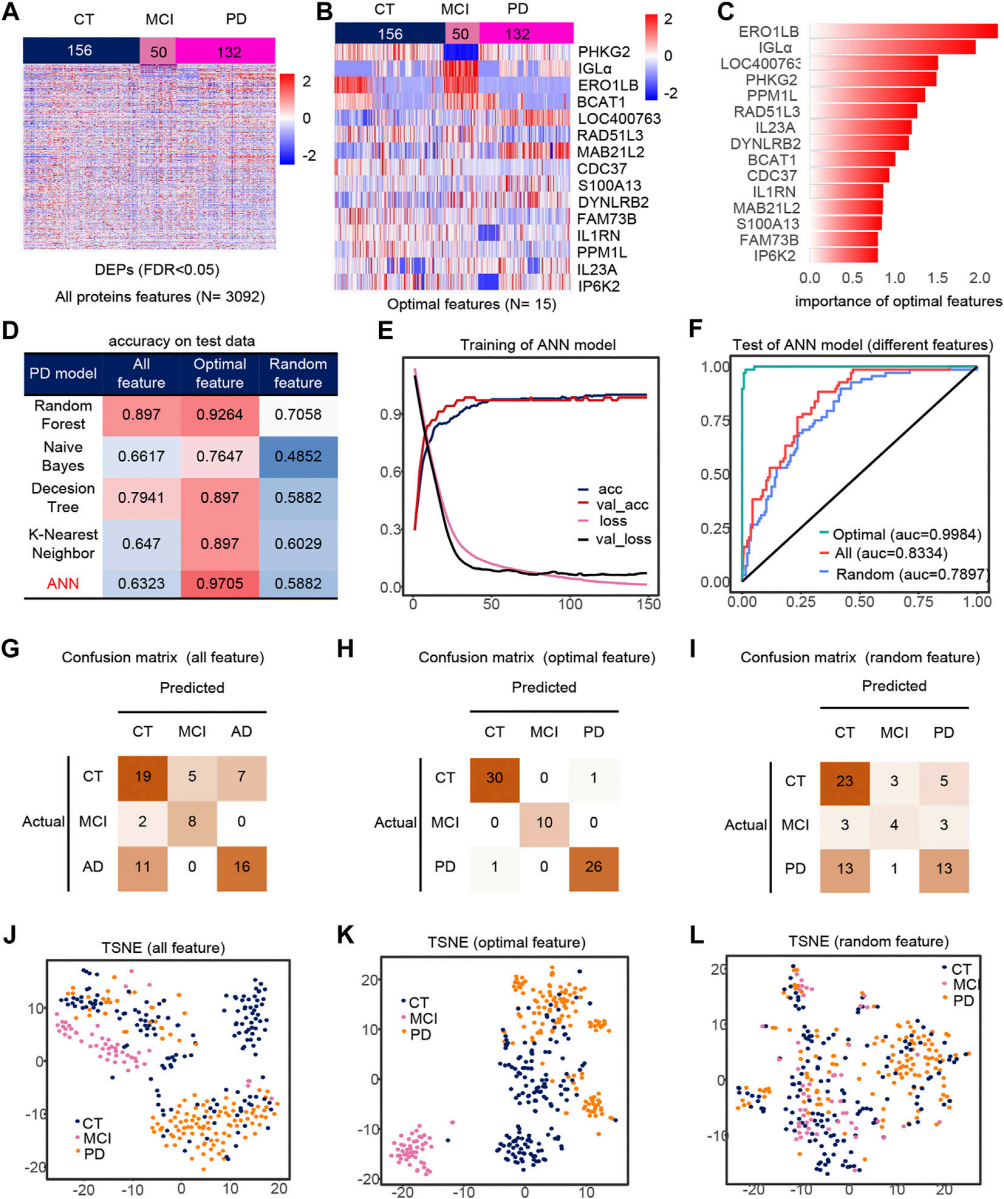
Model 2 (CT, MCI, PD) feature engineering and model evaluation. **(A)** Heat map of all features in the model. **(B)** Expression profile of the optimal features in the data set. c) Importance of the optimal features filtered after feature engineering. **(D)** Accuracy of different classifiers on different features. **(E)** Accuracy and loss curves during training of the ANN model. **(F)** AUC of the ANN model when using different features. **(G–I)** Confusion matrix corresponding to all features, optimal features, and random features in the ANN model. **(J–L)** TSNE consisting of all features, optimal features, and random features.

We extracted PD, CT and MCI samples from the dataset, using 3092 DEPs as initial features (Figure 3A). Finally, after feature selection, 15 features were retained (Supplementary Figures S1D,E), containing *ERO1LB*, *IGLa*, *LOC400763*, *PHKG2*, *PPM1L*, *RAD51L3*, *IL23A*, *DYNLRB2*, *BCAT1*, *CDC37*, *IL1RN*, *MAB21L2*, *S100A13*, *FAM73B*, *IP6K2*. Heat maps of the 15 features also showed significant differences between groups (Figures 3B,C). Similarly, the ANN model with feature engineering performed best (Figure 3D). When the model tends to be stable, the classification accuracy is the highest and the loss is the lowest (Figure 3E). The test set accuracy for the optimal features was 97.05%, where the MCI classification was completely accurate with micro-AUC of 0.9984, while all features were 0.83343 and random features were 0.7897 (Figures 3F,J–L). The accuracy of this model were greater than 0.94 in all three test sets (Figures 3G–I), and their AUCs in the test set are shown in the Supplementary Figures S4D–F. The optimal features distinguished the MCI samples well compared to all features and random features (Figures 3J–L). Finally, we also analyzed the correlation of optimal features with disease progression (Supplementary Figure S3F). Among these features, IL23a and IL1RN are pro-inflammatory cytokines and anti-inflammatory factors, respectively. MAB21L2 may be associated with neurodevelopment (Wang et al., 2020), and BACT1 knockout may cause oxidative neuronal damage (Mor et al., 2020).

### The AI Model for the Diagnosis of AD, PD, MCI and CT Based on 30 Serum Protein Markers

**FIGURE 4:**
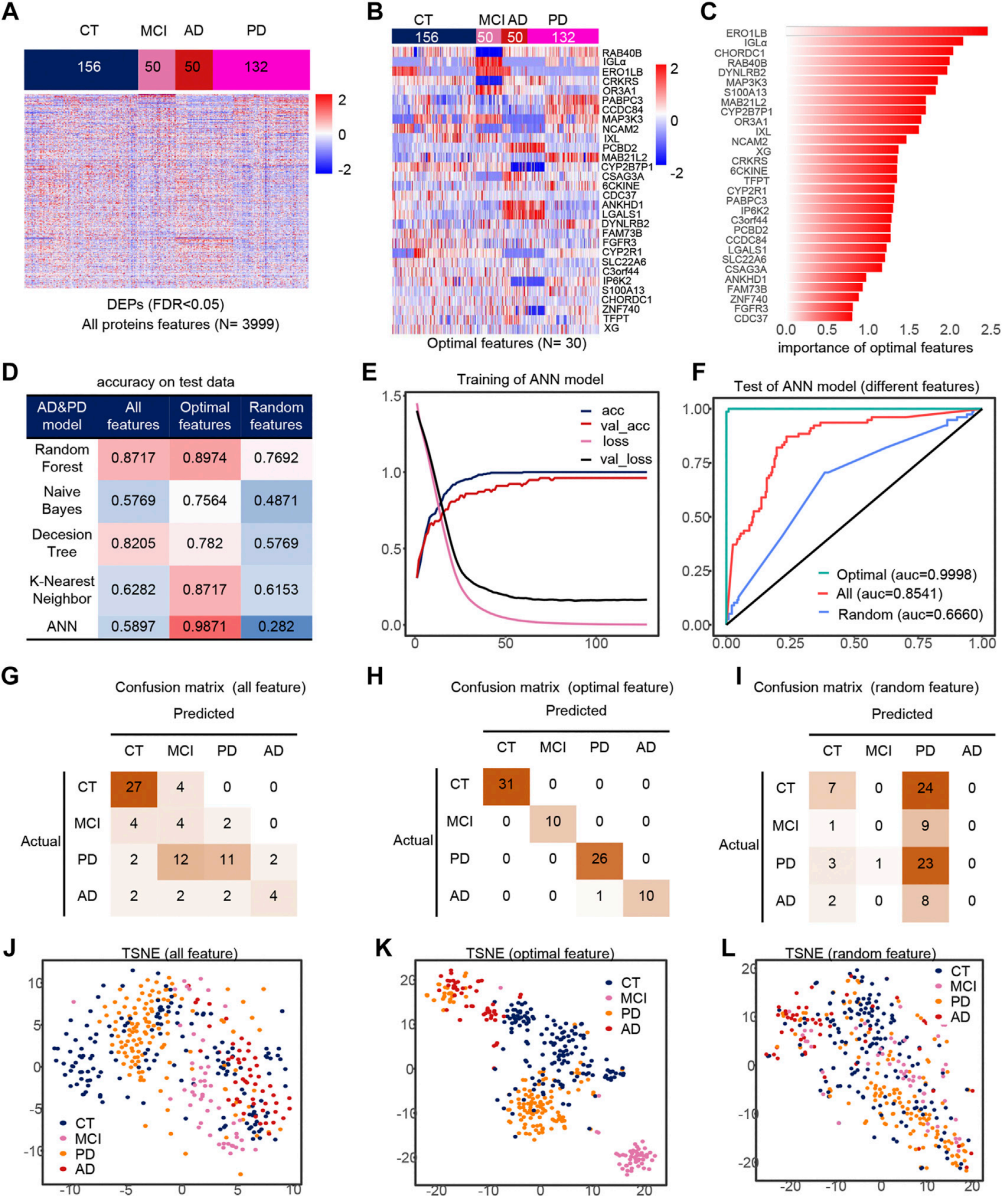
Model 3 (CT, MCI, AD, PD) feature engineering and model evaluation. **(A)** Heat map of all features in the model. **(B)** Expression profile of the optimal features in the data set. **(C)** Importance of the optimal features filtered after feature engineering. **(D)** Accuracy of different classifiers on different features. **(E)** Accuracy and loss curves during training of the ANN model. **(F)** AUC of the ANN model when using different features. **(G–I)** Confusion matrix corresponding to all features, optimal features, and random features in the ANN model. **(J–L)** TSNE consisting of all features, optimal features, and random features.

Similarly, we took out the DEPs of all samples for feature filtering and obtained the optimal model with 30 features (Figures 4A,B), where *PCBD2*, *LGALS1* belong to the features in model 1, while *IGLa*, *ERO1LB*, *MAB21L2*, *CDC37*, *DYNLRB2*, *FAM73B*, *IP6K2*, *S100A13* belong to model 2 features, which indicates that the features extracted by feature engineering have good robustness, and the importance of these 30 features is shown in the figure (Figure 4C). The filtered features are also optimal in the ANN model compared to other methods and other classifiers (Figure 4D). The accuracy of the model was greater than 0.95 in all three test sets (Figures 4G–I), and their AUCs in the test sets are shown in Supplementary Figures S4G–I. Compared to all features and random features, the optimal features outperform them and have a classification accuracy of 98.71% in all test sets, and all samples are correctly classified except one PD sample which is misclassfied as AD, and the micro-AUC reaches 0.9999 (Figures 4F,J–L), which is greater than 0.8541 for all features and 0.6660 for random features, and could fully identify MCI samples in the TSNE (Figures 4J–L).

### The Serum Proteins of Patients in the MCI, AD and PD Groups all Showed Different Differences From the Healthy Sample

In this paper, we first analyzed the DEPs of diseases. The number of DEPs in MCI, AD and PD compared to CT was 1010, 839 and 2122 respectively. The number of DEPs was 1221 and 1467 for AD and PD compared to MCI respectively. Finally, the number of DEPs between AD and PD was 2082 (Supplementary Figure S2A). First, PD was very different from CT, MCI, AD in terms of the number of DEPs, but was closest to MCI (Supplementary Figure S2B). Next, we found that the phase change proteins of MCI, AD, and PD were relatively different in location and cell type, with PD mostly distributed in the nucleus and enzymes, AD mostly distributed in the extracellular space and transcriptional regulators, and MCI mostly distributed in the cytoplasm (Supplementary Figures S2C,D). In addition, we found differences in phase separation scores in the cytoplasm between PD and normal samples, which may indicate that phase separation in PD is associated with the cytoplasm (Supplementary Figure S2E). Further analysis of the cell type scores in the cytoplasm suggests that the differences may lie in other cell types. Finally, we show the 10 proteins that differed most in disease relative to normal (Supplementary Figures S2G–I), with *EMG1*, *IFI6* are the most up-regulated and down-regulated DEP for MCI relative to normal, *ZCD2*, *IFI6* are the most up-regulated and down-regulated DEP for AD, and *CCT7*, *RANBP6* are the most up-regulated and down-regulated DEP for PD.

### Early PD May Occur Before Early MCI

**FIGURE 5:**
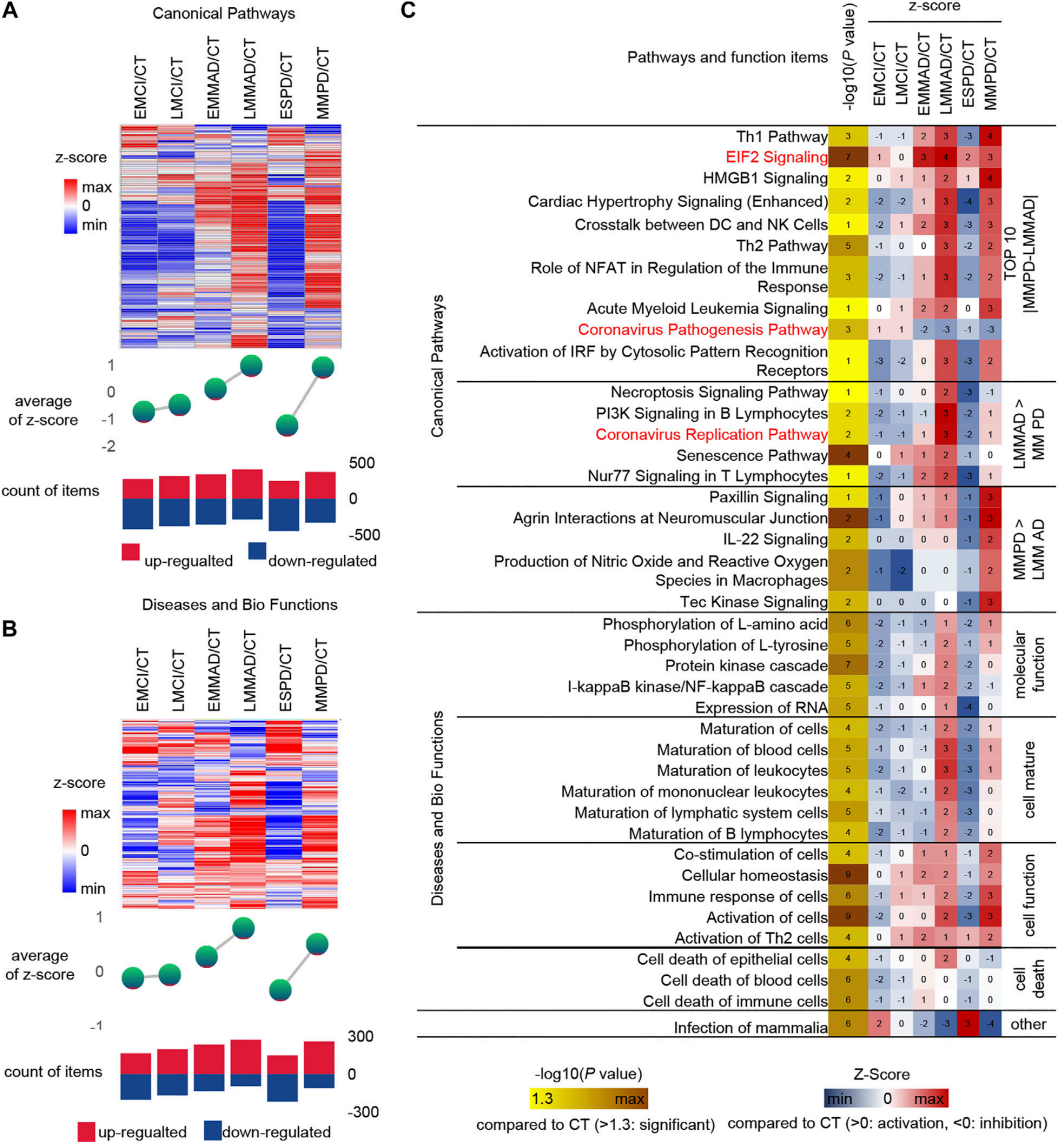
IPA analysis under detailed classification. **(A)** Expression, mean Z-score and the number of up-regulated and down-regulated of canonical pathways. **(B)** Expression, mean Z-score and the number of up-regulated and down-regulated of diseases and bio function **(C)** IPA analysis of Specific canonical pathways, diseases and biological functions of disease compared to CT group, respectively.

Serum molecules flow with the blood and can affect the body’s cells, tissues and organs in a comprehensive way. Regarding the biological events affected by serum molecules, we further analyzed the activation level of each biological event based on the conventional significance analysis. We classified the disease in more detail based on the underlying information, dividing MCI into early MCI (EMCI) and late MCI (LMCI), AD into early mild-moderate AD (EMMAD) and late mild-moderate AD (LMMAD), and PD into early PD (ESPD) and mild-moderate PD (MMPD). By observing the canonical pathways and disease and biological functions, we can find that the number of up-regulated pathways increased and the number of down-regulated pathways decreased during the process from EMCI/CT to LMMAD/CT (Figures 5A,B). Z-scores, the mean change in pathway relative to control samples, showed the same trend. ESPD followed the same trend as EMCI but with greater variability. The results show a continuum of inertia between multiple biological events in the organism of MCI and AD patients, while PD is more distinct from both. The incidence of biological events in the organism of patients with early PD was intermediate between that of healthy and early MCI. This suggests that early PD may precede early MCI.

Similarly, IPA was used for the analysis of seven groups of samples (Figure 5C). Among the canonical pathways, we selected the 10 pathways with the largest relative differences between MMPD and LMMAD. Prelonged activation of EIF2 leads to a sustained decrease in protein synthesis, which leads to memory impairment and neuronal damage (Halliday et al., 2017). The up-regulation ratio of EIF2 in MCI is small, while for AD and PD is larger, which may indicate that EIF2 is more related to neuronal damage.

Among the canonical pathways, we identified two pathways associated with the Coronavirus, namely the “Coronavirus Replication Pathway” and the “Coronavirus Pathogenesis Pathway.” Coronavirus have an enhanced replication capacity but reduced pathogenicity in disease compared to normal samples. The Coronavirus replication ability of AD is stronger than that of PD. It is known from the literature that patients with COVID-19 appear to be more susceptible to AD and that AD patients may be more susceptible to severe infection with COVID-19 (Ciaccio et al., 2021). In contrast, the current literature does not clearly indicate whether PD patients are more susceptible to COVID-19. This may reveal a greater susceptibility to COVID in AD.

The level of cell maturation is relatively low in the early stages of disease compared to normal samples, while in the middle and late stages of disease progression, cell maturation begins to increase abnormally to approaching or even exceeding normal levels. In terms of molecular function, excessive increases in the activating nuclear factor kappa B (NF-kB) have been shown to play an important role in driving Abeta deposition, neuroinflammation and neurodegenerative disease in AD, but NF-kB levels are not increased in PD, which may indicate that NF-kB does not promote α-synuclein (a-SYN) deposition (Lindsay et al., 2021).

### Possible Therapeutic Targets and Drugs

**FIGURE 6:**
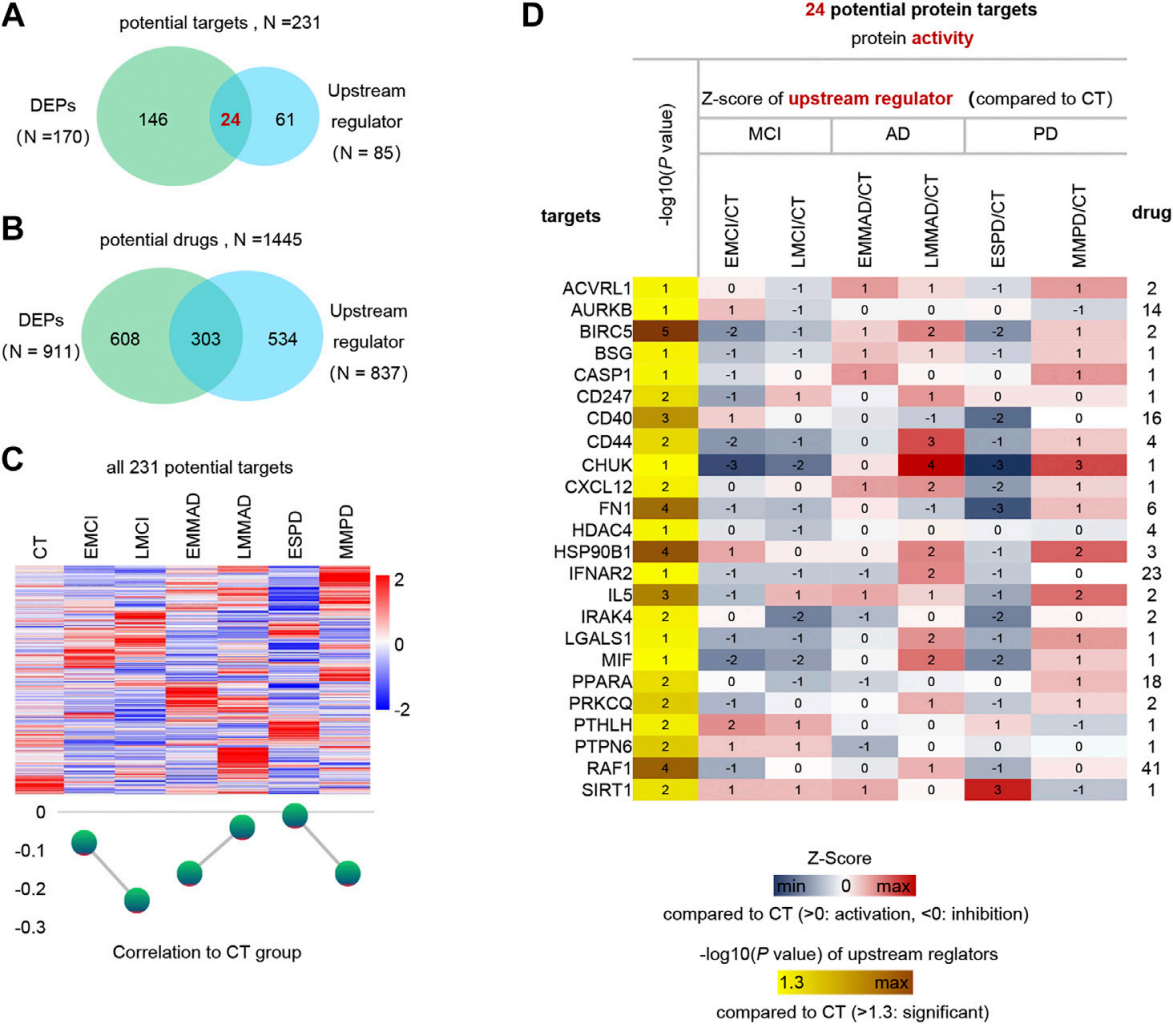
Analysis of upstream regulator and potential drugs of differentially expressed proteins. **(A)** Venn diagram of upstream regulators with drug and DEPs with drug. **(B)** Venn diagram of drugs corresponding to DEPs and drugs corresponding to upstream regulators. **(C)** Expression profiles of potential therapeutic targets in each group and mean Z-score. **(D)** Expression profiles of 24 differential upstream regulators.

Finally, we predicted the upstream regulators that may cause differences in protein profiles of patients. Upstream regulators of DEPs and corresponding drug treatment information were obtained by IPA annotation, of which 85 upstream regulators corresponding to 837 drugs. In addition, 170 DEPs corresponding to 911 drugs. These 231 kinds of DEPs and upstream regulators are potential therapeutic targets, and 1445 kinds of drugs can be considered as treatment options (Figures 6A,B). The expression of 231 potential targets in seven groups of samples is shown in Figure 6C. Among them, we can observe that ESPD is the closest to normal, which may also reflect the earlier onset of ESPD. Then, in order to further narrow the scope, we extracted 24 proteins that belong to both upstream regulators and DEPs. The predicted expression of these 24 upstream regulators is shown in Figure 6D and the corresponding drugs for all proteins are listed in Supplementary Table S1. In addition, machine learning models of LGALS1 were also present in 24 upstream factors. In the early stages of the disease, LGALS1 expression levels were reduced, along with reduced protein activity. In both AD and PD patients, LGLAS1 expression and protein activity were activated, which we speculate may be related to the overreaction of the organism. This may suggest the use of activators in the early stages and inhibitors in the late stages, and OTX008 is a target drug for LGALS1. We predict potentially intervenable drugs based on the activation levels of upstream factors, laying the foundation for diagnosis and intervention in neurodegenerative diseases such as AD and PD.

## Discussion

In neurodegenerative diseases, patients with MCI are a vague intermediate state that may not only present with early symptoms of AD and PD, but may also turn into normal. There is no good treatment for neurodegenerative diseases, and by the time they are diagnosed it is too late. Therefore, early diagnosis of neurodegenerative diseases is particularly important. It can help us have more time to think and cope with clinical symptoms before they appear.

Although there are lots of researches, many researchers’ models still have relatively large limitations. Zehra Karapinar Senturk uses voice data to identify PD samples and normal samples based on feature engineering and SVM classifiers. As a result, the classification accuracy is only 93.84% (KarapinarSenturk, 2020), which may be caused by feature engineering steps, i.e., filtering for importance only. In Jörn Lötsch’s study, a classifier was constructed using both olfactory and culinary information, and the machine learning model was able to discriminate non-PD samples with 94.1% accuracy, but only 58.9% for PD samples, which may be due to the extreme sample imbalance during model training (Lötsch et al., 2020). In Sanghee Moon’s study, which collected data from wearable devices and also used multiple data models, the highest accuracy was only 0.92, with the maximum f1 score was 0.61. In this study, the authors exposed the problem of sample imbalance, despite the simple feature engineering and oversampling methods (Moon et al., 2020). In Marek Wodzinski’s study, audio information was used, but the classification accuracy of the test set was only 0.90 (Wodzinski et al., 2019). More importantly, the above models are all binary models, which may lead to limited applications. In this paper, we combine bioinformatics methods and machine learning methods to filter out the important features in the data, i.e., we construct a reasonable feature engineering. The classifiers using this feature engineering can achieve higher accuracy, for example, all three classifiers in this paper have an accuracy of more than 97%. In addition, the features filtered by this method not only perform well on the model, but also the classification trend can be seen in a simple dimension reduction analysis. We use IPA method to analyze the upstream regulators (proteins, RNAs, drugs, metabolites, etc.) that form differential protein expression profiles and predict the activation or inhibition of their regulatory activities. Further, the proteins in the upstream regulators (activation or inhibition of protein activity) are selected and intersected with the differential expressed proteins (up- or down-regulation of expression abundance). Thus, the candidate targets and corresponding drugs are jointly identified from both protein activity and abundance perspectives.

We use IPA to analyze the activation level of each biological event based on between MCI, AD, PD and CT, and three of them deserve our attention, namely “Neuroinflammatory signaling pathway,” “JAK/Stat signaling pathway,” “Acute phase response signaling” (Supplementary Figure S3). Neuroinflammatory signaling is an immune response activated by microglia and astrocytes in the central nervous system (CNS), and is generally considered to be related to neurodegenerative diseases. The JAK/STAT pathway is a major signaling mechanism for several cytokines and growth factors (Murray, 2007). Inhibition of the JAK/STAT pathway may prevent neuroinflammation and neurodegeneration by suppressing the activation of a-SYN by innate and adaptive immune responses (Qin et al., 2016). In patients with AD and PD, the JAK/STAT signal pathway is activated and reversed in MCI, consistent with the neuroinflammatory pathway. In contrast to the traditional view that inflammation occurring in neurodegenerative diseases is chronic, IPA analysis believes that acute inflammation also occurs and plays an important role in neurodegenerative diseases. Previous studies have shown that the formation of senile plaques in patients with AD may involve acute inflammation (Sawada et al., 2015), and acute inflammation is also related to the severity of PD (Chen et al., 2012), which suggests that we need to re-examine the role of acute inflammation in neurodegenerative diseases. In addition, we feel that additional attention needs to be paid to the fact that early pd may appear before early mci in the IPA analysis, which may not be quite the same as what is perceived.

## Data Availability

Publicly available datasets were analyzed in this study. This data can be found here: Raw data can be download from the NCBI Gene Expression Omnibus under accession code GSE29654 (https://www.ncbi.nlm.nih.gov/geo/query/acc.cgi?acc=GSE29654), GSE62283 (https://www.ncbi.nlm.nih.gov/geo/query/acc.cgi?acc=GSE62283), and GSE74763 (https://www.ncbi.nlm.nih.gov/geo/query/acc.cgi?acc=GSE74763).

## Supplementary Material

Supplementary Figure S1 Variance and importance in feature engineering and the correlation between features and disease progression. **(A)** Filter the features with low variance in model1, the arrow indicates the 25% quantile of the overall variance, and filter the protein lower than the left side of the arrow. **(B)** The correlation between the features of model1. **(C)** The correlation between optimal features and disease progression in model1. **(D)** Filter the features with low variance in model2, the arrow indicates the 25% quantile of the overall variance, and filter the protein lower than the left side of the arrow. e) The correlation between the features of model2. **(F)** The correlation between optimal features and disease progression in model2. **(G)** Filter the features with low variance in mode3, the arrow indicates the 25% quantile of the overall variance, and the protein lower than the left side of the arrow is filtered. **(H)** The correlation between the features of model3.

Supplementary Figure S2 Overview of the DEGs. **(A)** The number of DEPs between different groups,the bar with light brown for normal vs. other samples, bar with light green for MCI vs. disease samples, and bar with light red for AD vs. PD. **(B)** Venn diagram of the number of DEPs for disease relative to normal. **(C)** Phase separation scores between different groups in cellular localization **(D)** Phase separation scores between different groups in cell type **(E)** Violin diagram of phase separation scores of different groups in cytoplasm. f) Phase separation scores between different cell types under cytoplasm **(G)** 10 most up-regulated and down-regulated DEPs for MCI relative to CT. **(H)** 10 most up-regulated and down-regulated DEPs for AD relative to CT. **(I)** 10 most up-regulated and down-regulated DEPs for PD relative to CT .

Supplementary Figure S3 Overview of the DEGs. **(A)** IPA analysis of canonical pathways, diseases and biofunctions of MCI, AD, PD compared to CT group, respectively.

Supplementary Figure S4 Overview of the DEGs. **(A)** AUC of each dataset in the model.

Supplementary Table S1 Expression trends of 24 therapeutic targets.

## Abbreviations

AD, alzheimer’s disease; AUC, area under curve; ANN, artificial neural network; CNS, central nervous system; CT, control samples; DEPs, differentially expressed proteins; DT, decision tree; EMCI, early MCI; EMMAD, early mild-moderate AD; ESPD, early PD; GEO, gene expression omnibus; IPA, Ingenuity Pathway Analysis; KNN, k-nearest neighbor; LMCI, late MCI; MCI, mild cognitive impairment; LMMAD, late mild-moderate AD; MMPD, mild-moderate PD; NB, naive bayes; NF-kB, nuclear factor kappa B; PD, parkinson’s disease; PDD, parkinson’s disease dementia; RLM, robust linear mode; RFm random forestl; a- SYN, α-synuclein.
